# Physiological Biodistribution of ^68^Ga-DOTA-TATE in Normal Subjects

**DOI:** 10.4274/mirt.galenos.2021.37268

**Published:** 2021-02-09

**Authors:** Salih Özgüven, Nuh Filizoğlu, Selin Kesim, Kevser Öksüzoğlu, Feyza Şen, Tunç Öneş, Sabahat İnanır, Halil Turgut Turoğlu, Tanju Yusuf Erdil

**Affiliations:** 1 Marmara University Pendik Training and Research Hospital, Clinic of Nuclear Medicine, İstanbul, Turkey

**Keywords:** 68Ga-DOTA-TATE, neuroendocrine tumors, PET/CT, somatostatin receptors, normal subjects

## Abstract

**Objectives:**

Somatostatin is an endocrine peptide hormone that regulates neurotransmission and cell proliferation by interacting with G protein-coupled somatostatin receptors (SSTRs). SSTRs are specific molecular targets of several radiotracers for neuroendocrine tumor (NET) imaging. Gallium-68 (^68^Ga)-DOTA-TATE is widely used for positron emission tomography/computed tomography (PET/CT) imaging of SSTRs and has shown a higher affinity for SSTR2, the most common SSTR subtype found in NETs. We aimed to analyze the distribution pattern of ^68^Ga-DOTA-TATE in normal subjects.

**Methods:**

A total of 617 consecutive ^68^Ga-DOTA-TATE PET/CT whole-body scans performed in our department from May 2015 through April 2020 with known or suspected neuroendocrine malignancies, mostly to evaluate adrenal adenomas, were retrospectively analyzed by 2 nuclear medicine physicians. One hundred eighteen subjects without a diagnosis of NET, with no tracer avid lesion of NET on ^68^Ga-DOTA-TATE PET/CT, and followed up for at least 6 months (average 2-3 years) without any biochemical, clinical, or imaging findings suggestive of NET were included in this study.

**Results:**

The highest uptake of ^68^Ga-DOTA-TATE was noted in the spleen followed by the kidneys, adrenal glands, liver, stomach, small intestine, prostate gland, pancreas head, pancreas body, thyroid gland, and uterus, in descending order. Minimal to mild uptake was detected in the submandibular glands, parotid glands, thymus, muscles, bones, breast, lungs, and mediastinum.

**Conclusion:**

Our study shows the biodistribution pattern of ^68^Ga-DOTA-TATE in normal subjects and the ranges of the maximum standard uptake value (SUV_max_) and SUV_mean_ values of 68Ga-DOTA-TATE obtained in several tissues for reliably identifying malignancy in ^68^Ga-DOTA-TATE PET/CT studies.

## Abstract (tr)

**Amaç:**

Somatostatin, G proteinine bağlı somatostatin reseptörleri (SSTR) ile etkileşerek endokrin sistemi, nörotransmisyonu ve hücre proliferasyonunu düzenleyen bir peptid hormonudur. SSTR’ler, nöroendokrin tümör (NET) görüntüleme için çeşitli radyoaktif madde işaretli spesifik moleküllerin hedefidir. Galyum-68 (^68^Ga)-DOTA-TATE, SSTR’lerin pozitron emisyon tomografisi/bilgisayarlı tomografi (PET/BT) görüntülemesinde yaygın olarak kullanılmaktadır ve NET’lerde bulunan en yaygın SSTR alt tipi olan SSTR2 için daha yüksek afinite gösterir.

**Yöntem:**

Mayıs 2015’ten Nisan 2020’ye kadar, çoğu adrenal adenomların değerlendirilmesi amacı ile olmak üzere, nöroendokrin malignitesi şüphesi olan veya NET tanılı olgulara bölümümüzde yapılan 617 ^68^Ga-DOTA-TATE PET/BT tüm vücut taraması, iki nükleer tıp hekimi tarafından geriye dönük olarak incelendi. NET tanısı olmayan, ^68^Ga-DOTA-TATE PET/BT’de aktivite tutulumu gösteren lezyon saptanmayan ve klinik, biyokimyasal veya görüntülemede NET bulgusu olmaksızın en az 6 ay (ortalama 2-3 yıl) izlenen yüz on sekiz olgu bu çalışmaya dahil edilmiştir.

**Bulgular:**

En yüksek ^68^Ga-DOTA-TATE tutulumu dalakta kaydedildi ve bunu sırasıyla böbrekler, adrenal bezler, karaciğer, mide, ince bağırsak, prostat bezi, pankreas başı, pankreas gövdesi, tiroid bezi ve uterus takip etti. Submandibular bezlerde, parotis bezlerinde, timusta, kaslarda, kemiklerde, memede, akciğerlerde ve mediastende minimal veya hafif tutulum tespit edildi.

**Sonuç:**

Bu çalışma, ^68^Ga-DOTA-TATE PET/BT görüntülemede maligniteyi güvenilir bir şekilde tanımlayabilmek için normal olgularda ^68^Ga-DOTATATE’nin biyolojik dağılım modelini ve çeşitli organlardan elde edilen maksimum standardize uptake değeri (SUV_maks_) ve SUV_ortalama_ değerlerinin aralıklarını gösterir.

## Introduction

Somatostatin is a peptide hormone that controls neurotransmission, hormone secretion, and cell proliferation by binding to somatostatin receptors (SSTRs). SSTRs are G protein-coupled membrane receptors presented on the cell surface of neuroendocrine cells. Five such receptor subtypes have been defined in humans (1,2). SSTRs are specific molecular targets of several radiotracers for neuroendocrine tumor (NET) imaging (3,4,5). However, the emergence of new positron emission tomography (PET) tracers has made PET/computed tomography (CT) imaging of SSTRs possible.

The somatostatin analogs Gallium-68 (^68^Ga)-DOTA-TOC (DOTA-Tyr^3^-octreotide), ^68^Ga-DOTA-NOC (DOTA-Nal^3^-octreotide), and ^68^Ga-DOTA-TATE (DOTA-Tyr^3^-octreotate) bind with varying affinity to SSTRs, and ^68^Ga-DOTA-TATE has shown higher affinity for SSTR subtype 2 (SSTR2) (6). The majority of gastroenteropancreatic NETs overexpress SSTR2, thus ^68^Ga-DOTA-TATE PET/CT is widely used to localize SSTR-expressing neuroendocrine neoplasms.

SSTRs are not only confined to NETs but are also demonstrated in various organs and hence, represent potential pitfalls. SSTR receptors have been described in the spleen, liver, pituitary gland, adrenal glands, head of the pancreas, thyroid, and urinary tract. It may be difficult to detect lesions in these organs, which show variable ^68^Ga-DOTA-TATE uptake (7). Therefore, it is crucial to know the biodistribution of ^68^Ga-DOTA-TATE when interpreting PET/CT imaging.

Recently, the number of studies outlining the role of ^68^Ga-DOTA-TATE PET/CT in the staging and management of NETs has increased (8,9,10,11); however, there are few studies in the literature that define the physiological uptake patterns of ^68^Ga-DOTA-TATE (7,12). In addition, there are limited data about the physiological uptake of ^68^Ga-DOTA-TATE in disease-free patients (13).

The objective of this study is to investigate the normal distribution pattern and physiological variants of ^68^Ga-DOTA-TATE in normal subjects on PET/CT imaging. This study presents the spectrum of normal standard uptake value (SUV) values in several organs and compares the results with previous reports. The main difference between this study and those previously reported is that our study population was proven to be clinically or pathologically disease-free before the examination and during follow-up.

## Materials and Methods

### Study Subjects

We retrospectively analyzed 617 consecutive ^68^Ga-DOTA-TATE PET/CT whole-body scans performed in our department from May 2015 through April 2020 on patients with known or suspected neuroendocrine malignancies, mostly to evaluate adrenal adenomas. One hundred eighteen subjects without a diagnosis of NET, with no tracer avid lesion of NET on ^68^Ga-DOTA-TATE PET/CT, and followed up for at least 6 months (average: 2-3 years) without any clinical, biochemical, or imaging evidence of NET were included in this study. Patients with a history or diagnosis of malignancy and younger than 18 years were excluded.

This study was performed with Marmara University Faculty of Medicine Research Ethics Committee review approval (date: December 2020, no: 09.2020.1317). All patients included in the study gave written informed consent before the examination.

### Preparation of 68Ga-DOTA-TATE

The ^68^Ga-DOTA-TATE was prepared on a fully automated system using a standardized labeling sequence. Briefly, a commercially available germanium-68 (^68^Ge)/^68^Ga generator (iThemba Labs, SA) was eluted with 0.6 M hydrochloric acid. Effluent containing the ^68^Ga fraction was transmitted to the PS-H+ cartridge to concentrate and purify ^68^Ga from residual ^68^Ge. The purified ^68^Ga was then eluted with 1.7 mL 5 M sodium chloride into the reaction vial. Twenty-five micrograms DOTA-TATE (ABX, Germany) was dissolved using 3 mL of 1.5 M HEPES buffer solution in the reaction vial. The reaction was performed at 100 °C for 8 minutes. A C-18 light cartridge was used to purify the ^68^Ga-DOTA-TATE. The purified ^68^Ga-DOTA-TATE was eluted with 1 mL ethanol and 1 mL water solutions and passed into a sterile vial. Radiochemical purity was over 95% in all cases, based on high-performance liquid chromatography.

### 68Ga-DOTA-TATE Imaging

All ^68^Ga-DOTA-TATE PET/CT scans were conducted using a hybrid PET/CT scanner (Discovery- 16 LS; GE Healthcare, Waukesha, Wisconsin, USA) in the Nuclear Medicine Department. Iohexol (Omnipaque; GE Healthcare) was used as the oral contrast agent. ^68^Ga-DOTA-TATE (2 MBq/kg) was administered intravenously. Whole-body images from skull base to mid-thigh were acquired 60±10 minutes after the injection. A low-dose 16-slice multidetector CT scan (parameters: 80 mA, 140 kV, table speed 27 mm/rotation, and slice width of 5.0 mm) was used to screen the body from mid-thigh to the base of the skull. A standard whole-body PET scan was conducted in 3D mode with an acquisition time of 3 min per bed position (six to eight bed positions) scanning the exact area with the CT scan. PET images were reconstructed with and without correction for attenuation using an iterative algorithm. Next, a workstation (Advantage Windows Workstation 4.6; GE Advantage) was used for processing and interpretation.

### Image Analysis

**Figure 1:**
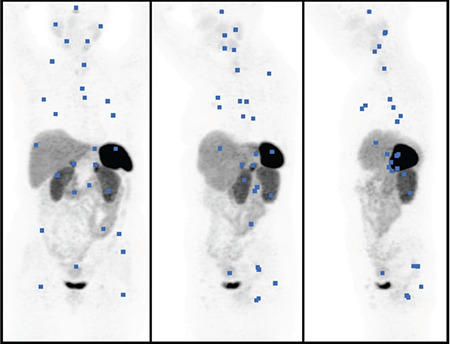
Regions of interest drawn on anterior, oblique, and lateral (from left to right) maximum intensity projection images

^68^Ga-DOTA-TATE PET/CT images were analyzed by two nuclear medicine physicians. Maximum SUV (SUV_max_) and SUV_mean_ values were calculated from a volume of interest (ROI) applied in the transaxial attenuation-corrected PET slice. ROIs obtained on CT images were applied to PET images. SUV_max_ was defined as the SUV_max_ in the ROI. SUV_mean_ was taken as the average SUV concentration in ROI. SUV_max_ and SUV_mean_ were evaluated on axial images in 29 normal anatomical structures for each patient using at least 2cm circular ROIs, avoiding inclusion of any activity from adjacent organs (Figure 1). Lung measurements were performed in the lower lobes away from the hilar vasculature, and kidney measurements were performed by avoiding the inclusion of pelvicalyceal urinary activity. SUV_max_ and SUV_mean_ values for the pituitary gland, parotid glands, submandibular glands, thymus, thyroid gland, mediastinum, lungs, breast, stomach, small intestine, liver, spleen, pancreas head, pancreas body, right adrenal gland, left adrenal gland, right kidney, left kidney, prostate, uterus, trapezius muscle, gluteal muscles, iliac crest, and femora were obtained. The maximum SUV_max_ and SUV_mean_ values are accepted as the representative values for that organ.

### Statistical Analysis

Univariate descriptive statistics [mean, median, standard deviation (SD), frequency, and range] were calculated on Microsoft Excel for Mac version 16.37 (Microsoft Corporation).

## Results

From our cohort of 118 subjects, 45 patients (38.1%) were men, and 73 patients (61.9%) were women. The average age of the patients was 51.83 years (range 18-85 years; SD 13.99 years). The SUV_max_ values were categorized as high, moderate, mild, and minimal in accordance with the study of Moradi et al. (7).

**Figure 2:**
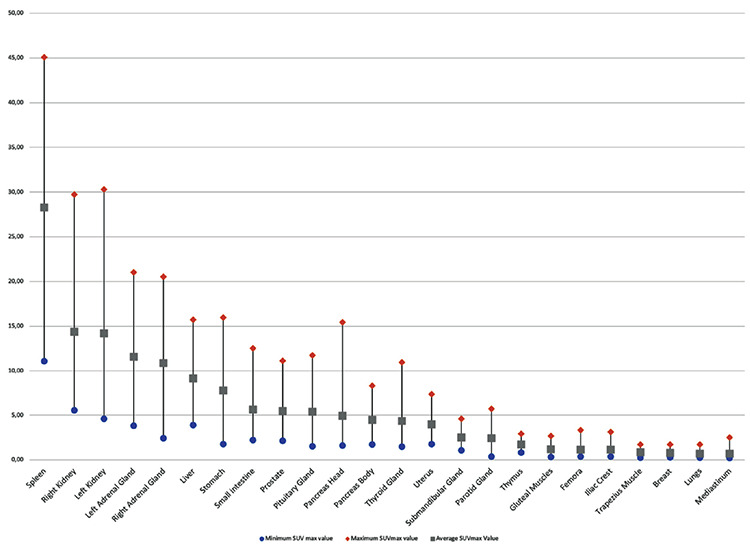
Average and range of maximum SUVs of different organs in normal subjects SUVs: Standard uptake values

Maximum physiological uptake was detected in the spleen. In addition to the spleen, high physiological uptake (average SUV_max_ >8.98 g/mL, which is the 50^th^ percentile of hepatic uptake) was also noted in the kidneys, adrenal glands, and liver, in descending order. Moderate uptake (average SUV_max_ >3.92, which demonstrated lower uptake than the liver) was observed in the stomach, small intestine, prostate gland, pancreas head and body, thyroid gland, and uterus. Mild uptake (from minimal uptake to moderate uptake) was revealed in the submandibular and parotid glands. Minimal uptake (average SUV_max_ <2 g/mL) of tracer was observed in the thymus (n=12), gluteal and trapezius muscles, femora, iliac crest, breast tissue, lungs, and mediastinum. No specific uptake (less than mediastinal blood pool activity) was seen in the subcutaneous fat tissue and brain tissue. The average SUV_max_ (± SD), average SUV_mean_ (± SD), and range of uptake on ^68^Ga-DOTA-TATE PET/CT for all the organs considered are summarized in Table 1. Figure 2, 3 represent the average and the range of physiological uptake of the organs as measured by SUV_max_ and SUV_mean_, respectively.

## Discussion

To the best of our knowledge, this is the first study to investigate the physiological distribution pattern of ^68^Ga-DOTA-TATE in normal subjects who had not previously been diagnosed with malignancy and who were proven to be clinically or pathologically disease-free during follow-up. This study also shows the ranges of the SUV_max_ and SUV_mean_ values of ^68^Ga-DOTA-TATE obtained in the different organs of normal subjects. The highest uptake of ^68^Ga-DOTA-TATE was documented in the spleen followed by the kidneys, adrenal glands, liver, stomach, small intestine, prostate gland, pancreas head, pancreas body, thyroid gland, and uterus, in descending order. Minimal to mild uptake was detected in the submandibular glands, parotid glands, thymus, muscles, bones, breast, lungs, and mediastinum.

In this study, when the distribution of ^68^Ga-DOTA-TATE was analyzed from the vertex to the mid-thigh, regarding the head region, moderate ^68^Ga-DOTA-TATE uptake in the pituitary gland was observed. This can be explained easily by the presence of SSTR2 in the anterior lobe cells of the pituitary gland (14). However, no activity uptake was observed in the cranium other than the pituitary gland. Although both SSTR1 and SSTR2 have been described in the cerebral cortex, the limbic system, the paraventricular nuclei of the hypothalamus and basal ganglia, ^68^Ga-DOTA-TATE cannot pass through the blood-brain barrier (15). Hence, ^68^Ga-DOTA-TATE uptake was not recorded in the brain parenchyma. The salivary glands, including the parotid and submandibular glands, demonstrated diffuse and homogenous uptake of ^68^Ga-DOTA-TATE, which is expected as Anzola et al. (16) demonstrated that SSTRs are commonly expressed in the salivary glands.

In the neck region, the thyroid gland showed wide variation in the uptake of ^68^Ga-DOTA-TATE in our study, and the ranges of SUV_max_ and SUV_mean_ were 1.48-10.97 and 0.81-5.93, respectively. SSTR2 expression in both normal and pathological thyroid tissues explain this situation. Thyroid adenomas, Grave’s disease, multinodular goiters, and active Hashimoto disease have been reported to increase the uptake of ^68^Ga-DOTA-TATE (17).

Since the glandular tissue of the breast expresses no significant SSTR2 (18), low levels of SUVs were observed in the breast. Unlike normal breast tissue, breast tumors may express different types of SSTRs (19). The risk of breast cancer should also be considered when focal and increased ^68^Ga-DOTA-TATE is detected.

In the chest, minimal ^68^Ga-DOTA-TATE uptake was observed in the lungs. SSTR2 is expressed on various components of lung inflammation, such as epithelial cells, inflammatory cells, and potentially fibroblasts (20). However, normal lung tissue mainly has SSTR4, which does not bind to ^68^Ga-DOTA-TATE, and in the absence of inflammation, lung tissue shows minimal uptake of ^68^Ga-DOTA-TATE, as in our study (20). Minimal ^68^Ga-DOTA-TATE uptake related to the mediastinal blood pool activity was also observed in our study. Adams et al. (21) showed the expression of SSTR1 and SSTR3 on inactivated endothelial cells, while SSTR2 is overexpressed on activated endothelial cells. Besides, granulocytes and red blood cells have no SSTRs (21). Therefore, only minimal uptake of ^68^Ga-DOTA-TATE in the mediastinum was detected in our normal subject group.

There are two primary components in the spleen, the red pulp and the white pulp. Studies have shown that SSTRs are primarily found in the red pulp of the spleen (22). Reubi et al. (23) also reported that red pulp comprises diffusely disseminated SSTRs. SSTR2 is the most frequent SSTR subtype presented in the spleen (24). As a result of this, the spleen showed intense ^68^Ga-DOTA-TATE uptake, resulting as expected in the highest SUV values, with average SUV_max_ and SUV_mean_ values of 28.27±5.99 and 19.25±4.36, respectively.

Relatively high ^68^Ga-DOTA-TATE uptake was also seen in the liver. The liver, which metabolizes peptides, is believed to extract ^68^Ga-DOTA-TATE from the blood, and this leads to hepatic uptake of ^68^Ga-DOTA-TATE (25). Furthermore, studies have shown that hepatocytes and hepatic stellate cells of the normal liver parenchyma do not express any of the SSTR subtypes (26). Although SSTR2 and SSTR4 are found in cholangiocytes and endothelial cells, we did not detect any ^68^Ga-DOTA-TATE activity in the biliary system.

Variable uptake of ^68^Ga-DOTA-TATE was detected throughout the pancreas. Higher physiological uptake in the uncinate process has been reported in previous studies due to the existence of subtypes 2, 3, and 5 of SSTR on islet cells and the higher density of islet cells in this region (27). However, Ionescu-Tirgoviste et al. (28) proved that the number of islets in the head of the pancreas is alike to that of other parts of the pancreas. In correlation with this, we observed similar SUV values in the head and body of the pancreas in our study (average SUV_max_ and SUV_mean_ were 4.94 and 2.90 for the pancreas head versus 4.46 and 2.87 for the pancreas body). Since islets may be present in clusters in any area of the pancreas, increased 68Ga-DOTA-TATE activity in such a region can be a normal variant for the pancreas.

A high uptake of ^68^Ga-DOTA-TATE was also found in the adrenal glands. The reason for this relatively high uptake is the presence of the five subtypes of SSTRs, mostly SSTR2, in the adrenal gland, which has been shown by Epelbaum et al. (29). SSTR2 expression in gastric cells has been demonstrated in previous *in vitro* immunohistochemistry studies (30). In correlation with these studies, we recorded high ^68^Ga-DOTA-TATE uptake in the stomach wall.

In our study, irregular and variable ^68^Ga-DOTA-TATE uptake was also observed in the intestine. It should be noted that this irregular and variable uptake may be the result of bowel motility and movement artifacts, as well as the expression of SSTR2 at different rates in the entire gastrointestinal (GI) tract. Previous studies have identified the SSTRs in the lymphoid tissue associated with the gut, myenteric, and submucosal plexus (30,31). Finally, the vessels in the inflammatory regions of the GI tract have been found to overexpress SSTR2, which can be another cause of focal uptake in the intestine (23).

In the evaluation of the urogenital system, the highest activity uptake was noted in the kidneys. DOTA peptide can be filtered through glomeruli but is also partially reabsorbed in the proximal tubule, which leads to increased activity besides the absorbed activity in the renal cortex (32). In the kidney, somatostatin lowers the glomerular filtration rate and reduces renal blood flow directly by renal vasoconstriction. It exerts an anti-diuretic effect by suppressing free water clearance at the cellular level and inhibiting vasopressin-induced water permeability in the distal tubules (33). Reubi et al. (34) demonstrated that vasa recta in the human kidney expresses high-density SSTR2. This could be the major reason for the high SUV values in the kidneys. SSTR2 receptors have also been shown in the tubular cells of the renal cortex, albeit at a lower density (34).

SSTRs have been found particularly in the stromal component of the prostate tissue. SSTR2 is preferably expressed in the normal prostate, while SSTR1 and SSTR5 are expressed in prostate cancer (35). SSTR2 deficiency in prostate cancer may explain the treatment ineffectiveness of some selective somatostatin analogs.

Green et al. (36) demonstrated SSTR2 expression in the endometrium during all stages of the menstrual cycle. Moreover, Schulz et al. (37) showed high SSTR1, SSTR2, and SSTR3 immunoreactivity in endometrial cancers. In line with these studies, a mild heterogeneous ^68^Ga-DOTA-TATE uptake in the uterus was seen in normal subjects.

A very low level of ^68^Ga-DOTA-TATE uptake in skeletal muscle and bones was observed. The reason for this minimal uptake is the expression of low levels of SSTRs in both osteoblasts and myoblasts. Therefore, a high level of ^68^Ga-DOTA-TATE uptake is not seen in the musculoskeletal system unless there is an inflammatory condition or malignancy (12).

### Study Limitations

This study had some limitations. For example, it included only subjects of Turkish nationality, so the results may not be generalized to populations of different ethnic origins. Another limitation is that our sample size was relatively small (n=12) to infer the range of normal SUV values of physiological thymic ^68^Ga-DOTA-TATE uptake.

## Conclusion

This study shows the biodistribution pattern of ^68^Ga-DOTA-TATE in normal subjects. The ranges of the SUV_max_ and SUV_mean_ values of ^68^Ga-DOTA-TATE obtained in the various organs is important for reliably identifying malignancy in ^68^Ga-DOTA-TATE PET/CT studies.

## Figures and Tables

**Table 1:**
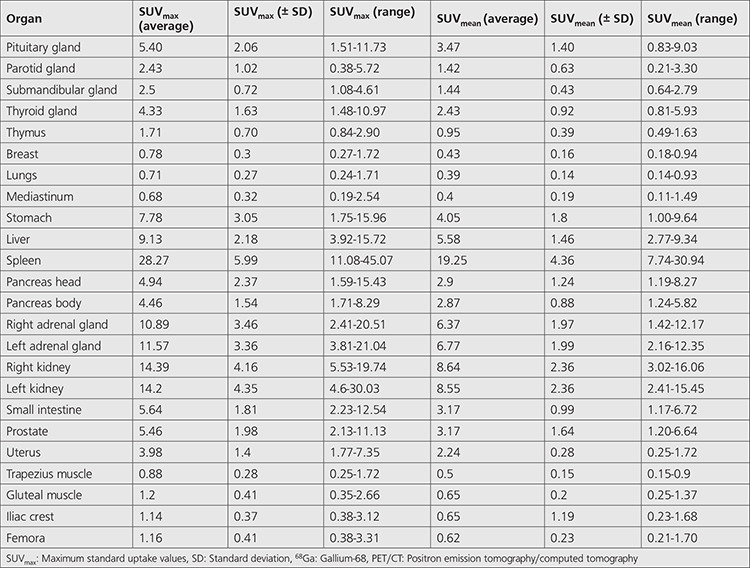
Average SUV_max_ (± SD), average SUV_mean_ (± SD), and range of uptake of ^68^Ga DOTA-TATE PET/CT for all organs

**Figure 3:**
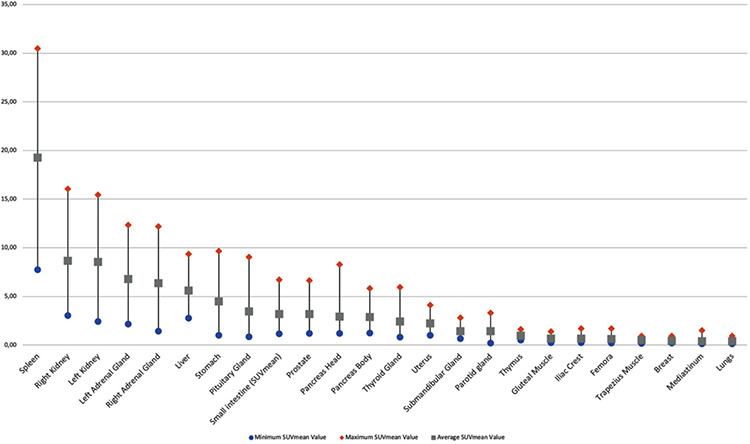
Average and range of mean SUVs of different organs in normal subjects SUVs: Standard uptake values

## References

[1] Bhanat E, Koch CA, Parmar R, Garla V, Vijayakumar V (2018). Somatostatin receptor expression in non-classical locations - clinical relevance?. Rev Endocr Metab Disord.

[2] Reubi JC, Waser B, Schaer JC, Laissue JA (2001). Somatostatin receptor sst1-sst5 expression in normal and neoplastic human tissues using receptor autoradiography with subtype-selective ligands. Eur J Nucl Med.

[3] Olsen JO, Pozderac RV, Hinkle G, Hill T, O’Dorisio TM, Schirmer WJ, Ellison EC, O’Dorisio MS (1995). Somatostatin receptor imaging of neuroendocrine tumors with indium-111 pentetreotide (Octreoscan). Semin Nucl Med.

[4] van den Anker-Lugtenburg PJ, Krenning EP, Oei HY, Van Hagen MP, Gerrits CJ, Reubi JC, Lamberts SW, Löwenberg B (1996). Somatostatin receptor scintigraphy in the initial staging of Hodgkin’s disease. Br J Haematol.

[5] Kwekkeboom DJ, Krenning EP (2002). Somatostatin receptor imaging. Semin Nucl Med.

[6] Virgolini I, Ambrosini V, Bomanji JB, Baum RP, Fanti S, Gabriel M, Papathanasiou ND, Pepe G, Oyen W, De Cristoforo C, Chiti A (2010). Procedure guidelines for PET/CT tumour imaging with 68Ga-DOTA-conjugated peptides: 68Ga-DOTA-TOC, 68Ga-DOTA-NOC, 68Ga-DOTA-TATE. Eur J Nucl Med Mol Imaging.

[7] Moradi F, Jamali M, Barkhodari A, Schneider B, Chin F, Quon A, Mittra ES, Iagaru A (2016). Spectrum of 68Ga-DOTA TATE Uptake in Patients With Neuroendocrine Tumors. Clin Nucl Med.

[8] Ilhan H, Fendler WP, Cyran CC, Spitzweg C, Auernhammer CJ, Gildehaus FJ, Bartenstein P, Angele MK, Haug AR (2015). Impact of (68) Ga-DOTATATE PET/CT on the surgical management of primary neuroendocrine tumors of the pancreas or ileum. Ann Surg Oncol.

[9] Mojtahedi A, Thamake S, Tworowska I, Ranganathan D, Delpassand ES (2014). The value of (68)Ga-DOTATATE PET/CT in diagnosis and management of neuroendocrine tumors compared to current FDA approved imaging modalities: a review of literature. Am J Nucl Med Mol Imaging.

[10] Gabriel M, Decristoforo C, Kendler D, Dobrozemsky G, Heute D, Uprimny C, Kovacs P, Von Guggenberg E, Bale R, Virgolini IJ (2007). 68Ga-DOTA-Tyr3-octreotide PET in neuroendocrine tumors: comparison with somatostatin receptor scintigraphy and CT. J Nucl Med.

[11] Al-Nahhas A, Win Z, Szyszko T, Singh A, Nanni C, Fanti S, Rubello D (2007). Gallium-68 PET: a new frontier in receptor cancer imaging. Anticancer Res.

[12] Kuyumcu S, Özkan ZG, Sanli Y, Yilmaz E, Mudun A, Adalet I, Unal S (2013). Physiological and tumoral uptake of (68)Ga-DOTATATE: standardized uptake values and challenges in interpretation. Ann Nucl Med.

[13] Shastry M, Kayani I, Wild D, Caplin M, Visvikis D, Gacinovic S, Reubi JC, Bomanji JB (2010). Distribution pattern of 68Ga-DOTATATE in disease-free patients. Nucl Med Commun.

[14] Shimon I (2003). Somatostatin receptors in pituitary and development of somatostatin receptor subtype-selective analogs. Endocrine.

[15] Sharma P, Mukherjee A, Bal C, Malhotra A, Kumar R (2013). Somatostatin receptor-based PET/CT of intracranial tumors: a potential area of application for 68 Ga-DOTA peptides?. AJR Am J Roentgenol.

[16] Anzola LK, Rivera JN, Dierckx RA, Lauri C, Valabrega S, Galli F, Moreno Lopez S, Glaudemans AWJM, Signore A (2019). Value of Somatostatin Receptor Scintigraphy with 99mTc-HYNIC-TOC in Patients with Primary Sjögren Syndrome. J Clin Med.

[17] Druckenthaner M, Schwarzer C, Ensinger C, Gabriel M, Prommegger R, Riccabona G, Decristoforo C (2007). Evidence for Somatostatin receptor 2 in thyroid tissue. Regul Pept.

[18] Albérini JL, Meunier B, Denzler B, Devillers A, Tass P, Dazord L, Le Simple T, Laissue J, de Jong R, Le Cloirec J, Reubi JC, Bourguet P (2000). Somatostatin receptor in breast cancer and axillary nodes: study with scintigraphy, histopathology and receptor autoradiography. Breast Cancer Res Treat.

[19] Dude I, Zhang Z, Rousseau J, Hundal-Jabal N, Colpo N, Merkens H, Lin KS, Bénard F (2017). Evaluation of agonist and antagonist radioligands for somatostatin receptor imaging of breast cancer using positron emission tomography. EJNMMI Radiopharm Chem.

[20] Gugger M, Waser B, Kappeler A, Schonbrunn A, Reubi JC (2004). Immunohistochemical localization of somatostatin receptor sst2A in human gut and lung tissue: possible implications for physiology and carcinogenesis. Ann N Y Acad Sci.

[21] Adams RL, Adams IP, Lindow SW, Zhong W, Atkin SL (2005). Somatostatin receptors 2 and 5 are preferentially expressed in proliferating endothelium. Br J Cancer.

[22] Melis M, Kaemmerer D, de Swart J, Kulkarni HR, Lupp A, Sänger J, Groen HC, Konijnenberg MW, de Jong M, Baum RP (2016). Localization of Radiolabeled Somatostatin Analogs in the Spleen. Clin Nucl Med.

[23] Reubi JC, Horisberger U, Kappeler A, Laissue JA (1998). Localization of receptors for vasoactive intestinal peptide, somatostatin, and substance P in distinct compartments of human lymphoid organs. Blood.

[24] Boy C, Heusner TA, Poeppel TD, Redmann-Bischofs A, Unger N, Jentzen W, Brandau W, Mann K, Antoch G, Bockisch A, Petersenn S (2011). 68Ga- DOTATOC PET/CT and somatostatin receptor (sst1-sst5) expression in normal human tissue: correlation of sst2 mRNA and SUVmax. Eur J Nucl Med Mol Imaging.

[25] Reynaert H, Rombouts K, Vandermonde A, Urbain D, Kumar U, Bioulac-Sage P, Pinzani M, Rosenbaum J, Geerts A (2004). Expression of somatostatin receptors in normal and cirrhotic human liver and in hepatocellular carcinoma. Gut.

[26] Reubi JC, Zimmermann A, Jonas S, Waser B, Neuhaus P, Läderach U, Wiedenmann B (1999). Regulatory peptide receptors in human hepatocellular carcinomas. Gut.

[27] Brabander T, Teunissen J, Kwekkeboom D (2017). Physiological Uptake in the Pancreatic Head on Somatostatin Receptor Scintigraphy Using [111In- DTPA]Octreotide: Incidence and Mechanism. Clin Nucl Med.

[28] Ionescu-Tirgoviste C, Gagniuc PA, Gubceac E, Mardare L, Popescu I, Dima S, Militaru M (2015). A 3D map of the islet routes throughout the healthy human pancreas. Sci Rep.

[29] Epelbaum J, Bertherat J, Prevost G, Kordon C, Meyerhof W, Wulfsen I, Richter D, Plouin PF (1995). Molecular and pharmacological characterization of somatostatin receptor subtypes in adrenal, extraadrenal, and malignant pheochromocytomas. J Clin Endocrinol Metab.

[30] Gugger M, Waser B, Kappeler A, Schonbrunn A, Reubi JC (2004). Cellular detection of sst2A receptors in human gastrointestinal tissue. Gut.

[31] Reubi JC, Horisberger U, Waser B, Gebbers JO, Laissue J (1992). Preferential location of somatostatin receptors in germinal centers of human gut lymphoid tissue. Gastroenterology.

[32] Hofman MS, Lau WF, Hicks RJ (2015). Somatostatin receptor imaging with 68Ga DOTATATE PET/CT: clinical utility, normal patterns, pearls, and pitfalls in interpretation. Radiographics.

[33] Balster DA, O’Dorisio MS, Summers MA, Turman MA (2001). Segmental expression of somatostatin receptor subtypes sst(1) and sst(2) in tubules and glomeruli of human kidney. Am J Physiol Renal Physiol.

[34] Reubi JC, Horisberger U, Studer UE, Waser B, Laissue JA (1993). Human kidney as target for somatostatin: high affinity receptors in tubules and vasa recta. J Clin Endocrinol Metab.

[35] Reubi JC, Waser B, Schaer JC, Markwalder R (1995). Somatostatin receptors in human prostate and prostate cancer. J Clin Endocrinol Metab.

[36] Green VL, Richmond I, Maguiness S, Robinson J, Helboe L, Adams IP, Drummond NS, Atkin SL (2002). Somatostatin receptor 2 expression in the human endometrium through the menstrual cycle. Clin Endocrinol (Oxf).

[37] Schulz S, Schmitt J, Weise W (2003). Frequent expression of immunoreactive somatostatin receptors in cervical and endometrial cancer. Gynecol Oncol.

